# Women’s empowerment and gender equality in South Asian agriculture: Measuring progress using the project-level Women’s Empowerment in Agriculture Index (pro-WEAI) in Bangladesh and India

**DOI:** 10.1016/j.worlddev.2021.105396

**Published:** 2022-03

**Authors:** Agnes Quisumbing, Ruth Meinzen-Dick, Hazel Malapit

**Affiliations:** International Food Policy Research Institute, Washington, DC, USA

**Keywords:** Women’s empowerment, Gender equality, Agricultural development programs, South Asia, Bangladesh, India

## Abstract

This introduction to a special section describes how a recently developed measure, the project-level Women’s Empowerment in Agriculture Index (pro-WEAI) can be used to assess empowerment impacts of agricultural development interventions in India and Bangladesh as well as broader changes in rural labor markets. The special section comprises three papers. The first examines the impact of membership in self-help groups in five states in rural India on women’s and men’s empowerment and gender equality. The second presents experimental evidence from a pilot project in Bangladesh that provided trainings in agricultural extension, nutrition behavior change communication, and gender sensitization to husbands and wives together. The third investigates changes in women’s roles within the jute value chain in the Southern Delta region of Bangladesh as household members migrate out of the study area and the availability of male labor declines. Although these papers focus on Bangladesh and India, pro-WEAI can be applied to impact assessments of agricultural development projects more generally. The three papers show both the usefulness of this new measure in detecting changes in empowerment indicators within the lifespan of a project and the value of having explicit empowerment objectives in agricultural development projects. The papers also demonstrate the value of having data on both men and women so that project designers can be more intentional about including both of them and monitoring outcomes for both to promote more gender equitable outcomes.

## Highlights

• Three studies in India and Bangladesh use pro-WEAI to examine empowerment impacts of agricultural interventions and labor market changes.
• Pro-WEAI shows potential in detecting empowerment impacts within the project lifespan.
• Findings suggest the need for intentionality in designing women’s empowerment projects.
• It is important to collect data on both women and men to track progress toward both women’s empowerment and gender equality.

## 1 Introduction

There is an emerging consensus within the international development community that gender equality and women’s empowerment are important goals from a human rights perspective, as well as for achieving a range of economic and social development objectives such as improved food security, child nutrition and education, and women’s health (Johnson et al., 2018). Increasingly, policymakers and practitioners have recognized the importance of women’s empowerment in increasing agricultural productivity and reducing poverty. Accordingly, many organizations have incorporated empowerment objectives and integrated activities designed to empower women into their agricultural projects and programs. However, to be able to monitor progress toward achieving these goals, reliable metrics of women’s empowerment and gender equality are needed.

Measuring women’s empowerment requires understanding what it is. We use Kabeer’s (1999) definition of empowerment as a process that expands people’s ability to make strategic life choices, particularly in contexts in which this ability had been denied to them. Measures such as the Gender Gap Index (World Economic Forum (2018) and previous years), the Gender Development Index (GDI), and the Gender Inequality Index (GII) (UNDP, 2018) that rely on administrative or aggregate data have focused on gender equality, rather than women’s empowerment. However, indices that only look at equality are incomplete, because equality is relative. Worsening men’s indicators would improve equality even if women’s indicators do not improve, whereas one should be interested in improving both women’s empowerment and gender equality.

More recent efforts measure women’s empowerment directly and interview women themselves, rather than use proxy indicators from administrative data. For example, the Women’s Empowerment in Agriculture Index (WEAI) (Alkire et al., 2013), the Women’s Empowerment in Livestock Index (WELI) (Galie et al., 2018), and the Women’s Empowerment in Nutrition Index (WENI) (Narayanan et al. 2019). are based on interviews of women themselves, and in some cases, like the WEAI and WELI, interviews of men within the same households, to also assess gender parity. Further work has been undertaken to adapt the WEAI for specific uses. An abbreviated version of the WEAI (A-WEAI) was developed to reduce interview time and eliminated some modules that were difficult to implement in the field (Malapit et al. 2017). However, both the WEAI and A-WEAI were intended for population-based surveys and none, with the exception of WELI, were specifically designed for monitoring progress towards women’s empowerment in a project implementation setting.

In response to the demand from agricultural development projects, the Gender, Agriculture, and Assets Project, Phase 2 (GAAP2) worked with 13 agricultural development projects to develop a project-level WEAI (pro-WEAI) that is suited for use in project impact evaluations. This meant refining indicators to be more sensitive and responsive to project effects within the typical time frame of project implementation, as well as adding indicators that participating projects felt were important for gauging project success (for example, indicators related to gender-based violence against women, mobility, and intrahousehold harmony) (Malapit et al. 2019). Similar to the WEAI, pro-WEAI draws on Kabeer’s (1999) definition of empowerment as encompassing agency, resources, and achievements, but focuses more closely on agency. Pro-WEAI includes indicators in three domains of agency: intrinsic agency, instrumental agency, and collective agency. These measures can also be used outside the project setting to examine empowerment impacts of processes related to structural transformation, such as increasing market orientation or migration.

The three papers in this special section come from three GAAP2 partner projects in India and Bangladesh that measure empowerment using pro-WEAI. They illustrate how pro-WEAI and its component indicators can be used to examine changes in women’s empowerment and gender equality arising from nutrition- and gender-sensitive agricultural development projects as well as broader changes in the labor market, such as migration and employment.

The first paper by Kumar et al. examines the potentially empowering effects of membership in women’s groups. In the past three decades, women’s groups have rapidly gained prominence as important rural financial and social institutions in South Asia. Their role has expanded to include creating health and nutrition awareness, generating demand for government programs, ensuring transparency in the implementation of government schemes and tackling social issues ranging from dowry and domestic violence to gender and caste-based discrimination. In India, a large majority of women’s group-based programs are implemented through self-help groups (SHGs).

Kumar et al. examine the impact of SHG membership on empowerment outcomes, drawing from data collected in an evaluation of a nutrition-sensitive agricultural intervention being implemented by PRADAN, one of India’s largest NGOs, using its women’s self-help groups platform. The main evaluation focuses on the impact of a nutrition-sensitization arm layered onto PRADAN’s existing agricultural extension platform; the analysis presented here looks at SHG membership in both the treatment and comparison arms and includes women who are members of non-PRADAN SHGs. The SHG model includes training sessions on women’s empowerment and political participation, and therefore is expected to have impacts on aspects of women’s empowerment. While a recent systematic review shows that women’s SHGs have positive effects on economic and political empowerment (Brody et al., 2017), there is limited evidence on their effect on women’s empowerment in agriculture and no evidence on how they affect men within these households. Understanding the impact of SHG membership on women’s empowerment in agriculture is important in the Indian context because of the scale of the SHG platform (SHGs cover approximately 48 million households), the size of the agricultural sector, and the extent of discrimination against women.

Kumar et al. use panel data of 1470 women from five states in India to study the impact of SHG membership on women’s empowerment in agriculture. The paper uses nearest neighbor matching to identify the impact of SHG membership on women’s empowerment outcomes. The results suggest that SHG membership increases women’s empowerment scores and reduces the gap between men’s and women’s empowerment scores within a household. This improvement in aggregate empowerment is driven by improvements in women’s scores, not a deterioration in men’s. Compared to similar nonmembers, SHG members have greater control over income, credit access and participation in credit decisions (areas of instrumental agency) and are more likely to be active members of at least one group but have higher workloads. SHG membership, however, affects neither respect among household members nor tolerance towards domestic violence, aspects of intrinsic agency that may be more difficult to affect. The authors conclude that SHG membership can empower women in domains related to agriculture, but long-standing gender norms may take longer to change.

The PRADAN SHG model, like other SHG programs in India, targets its programming only to women and does not involve men. What are the empowerment impacts of gender- and nutrition-sensitive agricultural projects that target both women and men?

The paper by Quisumbing et al. presents results from Agriculture, Nutrition, and Gender Linkages (ANGeL), a pilot project in Bangladesh that engaged with both men and women. ANGeL was implemented by the Ministry of Agriculture and assessed three intervention components for promoting nutrition- and gender-sensitive agriculture through group-based trainings for husbands and wives together: (1) agricultural extension, (2) nutrition behavior change communication (BCC), and (3) gender sensitization. The project was a randomized controlled trial that examined the impacts of these components alone and bundled together, with the following treatment arms: only agricultural extension (T-A); only nutrition BCC (T-N); bundled agricultural extension and nutrition BCC (T-AN); and bundled agricultural extension, nutrition BCC, and gender sensitization (T-ANG). The design allowed one of the first experimental evaluations of the impact of a gender-sensitive agriculture-nutrition intervention on empowerment and related outcomes. For women, all treatment arms significantly improved empowerment scores and empowerment status, driven by increases in instrumental agency, with no significant difference across arms. For men, no treatment arm reduced empowerment, suggesting that women’s empowerment gains did not come at men’s expense, and in fact T-N significantly improved men’s empowerment scores and status in terms of instrumental agency. T-AN and T-ANG increased gender parity and improved women’s gender attitudes; T-N, T-AN, and T-ANG improved men’s gender attitudes, particularly those related to husbands helping wives with household chores. In terms of unintended impacts, there were no significant effects on workloads, while T-ANG showed inconclusive effects on intimate partner violence. Overall, the study suggests potential benefits of bundling components within an agricultural development intervention; however, many of these benefits seem to be driven by bundling nutrition with agriculture. Adding the gender sensitization training did not have a significantly greater impact on women’s empowerment or gender parity than the other modalities. The fact that all treatment arms trained men and women together may have contributed to the positive outcomes—an interpretation supported by the qualitative data. Results suggest that intentionality in project design can contribute to making an agricultural project gender-transformative. Rather than explicit focus on gender-related content, ANGeL’s delivery platform may have played an important role – training men and women together on topics within each other’s traditional domain.

Finally, the paper by de Brauw et al., written in the context of a value chain development intervention, studies how women’s roles within the jute value chain in the Southern Delta region of Bangladesh change as household members are migrating out of the study area, and the availability of male labor declines. It also examines whether outmigration and changing labor availability is associated with women’s empowerment and gender wage gaps and provides broader recommendations for analyzing gendered outcomes in panel and event studies. Using a panel sample of 1500 smallholder households from 50 villages, the surveys use pro-WEAI to measure women’s empowerment. The authors also collected, by stage of jute production, the amount of male and female *household* labor used, the amount of male and female *hired* labor used, and any wages paid to these male and female laborers, which are used to measure gender wage gaps.

In producer households, de Brauw et al. find low rates of women’s empowerment, driven by a lack of women’s autonomy in jute production and a high workload outside of agriculture. They also observe strong gender differentiation in tasks, with women in jute production participating mainly in post-harvesting tasks, which take place within the family compound. Although producer households use more female household labor as they experience increased labor scarcity, and pay higher wages to male laborers, they do not hire additional female labor; and neither women’s empowerment nor female wages improve when male labor becomes scarce. Consequently, in the context of existing gender norms, women face an increased workload and a widening gender wage gap in a context of increasing male labor scarcity, suggesting limited opportunities to empower women through the jute value chain and labor market. However, women’s empowerment improves absolutely and compared to men’s empowerment, when other female household members migrate out. The authors conclude that more work is needed to identify innovations that allow women to take advantage of new opportunities in rural labor markets, generated in a context of rapid urbanization, in order for them to become more empowered within agricultural value chains.

The pro-WEAI was originally designed for project impact assessments. The papers on the impact of SHG membership and the evaluation of the ANGeL project show that the index can capture changes in empowerment status that are attributable to project interventions. Beyond the individual studies, this special section is the first to bring together different impact assessments to show how an index drawing on a counting-based measurement approach, for which the definitions, thresholds, and weights of each indicator are explicitly defined (Alkire et al., 2015), can provide insights on how certain interventions affect empowerment. Comparing across the projects we note that two of the three—PRADAN’s SHGs and ANGel—were intentional about women’s empowerment in their program content and delivery mechanisms (e.g. SHGs or training men and women together). It was these projects that had an empowerment effect, not only in the areas of direct intervention (e.g. group membership in the case of SHGs), but on broader aspects, such as control over income and financial inclusion (credit access and participation in credit decisions). However, not all indicators of empowerment necessarily move together; the multiple indicators allow us to check for unintended consequences, such as increased workloads. Comparison of the pro-WEAI and A-WEAI in these two studies shows that, because it has more indicators (e.g. on intrinsic agency) and higher thresholds, pro-WEAI is more sensitive to changes within the project life span, which is useful for impact assessment.

The value chain development intervention analyzed by De Brauw et al. originally had program components addressing gender, but over time, because of changes in programming emphasis, these were dropped from the jute value chain activities studied in the impact evaluation. As could be expected, the evaluation team found that there were no empowerment impacts (de Brauw et al., 2018). However, other trends in the project area, such as male outmigration, had important gendered impacts. The paper by de Brauw et al. illustrates that pro-WEAI can be used to analyze empowerment effects of broader trends in rural transformation, such as migration and participation in labor markets, which can shape opportunities through which future program components can enhance women’s empowerment. Ultimately, because the data are collected at the individual and household level, it is possible to test for the interaction between project interventions and broader trends such as migration, or other characteristics of program participants. The papers in this special section suggest that, while agricultural development projects have the potential to empower women and reduce gender disparities, other factors such as broader labor market dynamics and cultural norms also affect the status of women and intrahousehold relations.

While the emphasis in these studies is on women’s empowerment, they demonstrate the value of having data on men as well as women. Not only does this allow us to examine factors affecting intrahousehold gender parity, the results indicate that interventions targeting women can also contribute to men’s empowerment (e.g. on financial inclusion). Data on men’s empowerment enable project designers and implementors to identify where men are also disempowered and pay attention to possible backlash if an intervention inadvertently disempowers men. More importantly, having data on men enables project designers to be more intentional about including both women and men in interventions and measuring impacts on both their outcomes to be able to transform harmful gender norms and dynamics and promote more gender equitable outcomes.
